# The study of etiological and demographic characteristics of acute household accidental poisoning in children - a consecutive case series study from Pakistan

**DOI:** 10.1186/1471-2431-10-28

**Published:** 2010-05-03

**Authors:** Nabeel Manzar, Syed Muhammad Ali Saad, Bushra Manzar, Syeda Shahzeen Fatima

**Affiliations:** 1 Department of Pediatrics, Dow University Of Health Sciences, Karachi, Pakistan

## Abstract

**Background:**

To determine the agents of poisoning and demographic distribution of children brought to Civil Hospital Karachi (CHK) with a history of accidental poison intake and to examine the factors associated with it.

**Methods:**

This hospital based descriptive study of first 100 patients from both sexes who presented to Pediatric department, CHK from 1^st ^January 2006 till 31^st ^December 2008 with exposure to a known poisonous agent and fulfilling other inclusion criteria were included in the study. Data regarding their demographic profile and potential risk factors was collected on a well structured proforma, cases were followed until discharge or expiry. Data was analyzed using frequencies, proportions, group means, median and standard deviations.

**Results:**

The male to female ratio in our study was 1.2:1, with kerosene (50%) being the most common household agent followed by medicines (38%), insecticides (7%) and bathroom cleaners (5%). Factors such as mother's education level, number of siblings and storage place of poison correlated significantly with the cases of accidental poisoning. Most of the children (70%) presented within 3 hours of ingestion. Dyspnea was the most common symptom observed. The mortality rate in our study was 3%.

**Conclusions:**

Children belonging to age group 2-3 years are the most susceptible both in terms of morbidity and mortality. Preventive strategies need to be adopted at a national level to spread awareness among parents.

## Background

Among the leading causes of injury, poison as a form of injurious agent stands 2^nd ^with falls being the first and burns, drowning following on. Poisoning has also been the 3^rd ^most common injury treated in emergency rooms for children less than 16 years of age[1]. A study carried out in National Institute of Child Health, Pakistan in 1988 noted that 2.98% patients coming to casualty were with some forms of poisoning[2].

Accidental poisoning is a problem of huge magnitude [3] and a persistent cause of injury related morbidity and mortality worldwide. In 1940 childhood poisoning was responsible for an estimated 500 deaths per year due to primarily some household products[4]. This is the 21^st ^century and we have a come a long way in combating accidental poisoning with the establishment of first poison control centre in 1953 in Chicago, USA. However, in developing countries like Pakistan accidental household poisoning continues unabated due to lack of functional poison control centers and the lack of awareness among the masses.

New research indicates that various social and demographic factors like family size, socioeconomic condition, attention to child as well as storage place of poison are important risk factors which significantly influence the acute household poisoning cases in children[5,6]. There is a lot of data and research about accidental childhood household poisoning from developed countries, [7-9] however, there is a paucity of data from Pakistan because there is neither a national database nor any relevant authority but individual studies have been carried out in local cities [10,11] in the past. The magnitude of the problem as well as significant morbidity and mortality, parent's anguish and cost of admission and treatment incurred to the state compelled us to carry out our research. Factors such as patient's age, poisoning agent, demographic distribution, parents' education level especially of mothers, number of siblings, presenting symptoms and their outcome in terms of hospital stay and mortality were taken into consideration. The main objective of our study was to determine the agents of poisoning and demographic distribution of children brought to CHK with a history of accidental poison intake and to examine the various risk factors associated with it.

## Methods

This hospital based, retrospective and non-interventional case series study was carried at Pediatrics Emergency Care Department, CHK, Pakistan. Patient data files were analyzed starting from the period of 1^st ^January 2006 till 31^st ^December 2008. First 100 patients diagnosed with accidental household poisoning with different agents and who fulfilled the inclusion criteria were included in the study. The study protocol was reviewed and approved by the ethics committee at the study centre (Dow University of Health Sciences-Ethical Review Committee) and the study was carried out in accordance with the declaration of Helsinki of 1975, revised in 1983.

Patients fulfilling the following study criteria were enrolled in the study:

1) Subjects brought alive to emergency room of pediatric department

2) Patients of aged up to 12 years with established diagnosis of acute household poisoning.

3) Known household poisonous agent.

4) Informed consent from subjects or family for participating in the study.

Patients with the following conditions were excluded from the study:

1) History of snake bite, dog bite, insect sting.

2) Non-accidental poisoning i.e. suspected suicidal or homicidal poisoning.

3) Definitive history of ingestion of household substance not available.

4) Children having history of poison exposure through other routes like ocular or nasal as well as with any other concurrent illness like diarrhea, vomiting, renal or liver disease.

The study was carried out in two parts, first all patients had their detailed medical history taken with complete physical examination followed by data collection by the investigators on a well structured proforma regarding their social and demographic characteristics. Any complication that occurred during stay in hospital was also recorded. Cases were followed until discharge or expiry of patient.

Classification of subjects was done according to age, sex, residence, time lapse between ingestion and presentation, potential risk factors and outcome criteria. Subjects were divided into two age groups (1) = 2 years in age (2) >2 years. Time duration between ingestion of known substance and presentation was divided into two groups (1) = 180 minutes (2) >180 minutes. The subject's area of residence was also classified into urban and rural areas depending on whether they belonged to the city of Karachi or villages outside Karachi. The potential risk factors were also classified accordingly (Table. 3)

Data was entered in Statistical Package for Social Sciences (SPSS version 12) and all the data was analyzed retrospectively. Data for baseline characteristics of patients was expressed as means ± standard deviation (S.D). In our study all the data was collected on medical checkups. Analysis of the data was done using descriptive statistics like frequencies, proportions, group means, median and standard deviations.

## Results

There were 54 males and 46 females in the study. The male to female ratio was almost 1:1. The mean (standard deviation) age of all these children was 34.9 (13.0) with median of 36 months. Most of the children (66%) were above 2 years old. Eighty-five percent of the children in the study were living in urban areas. Fifty children had history of kerosene oil intake while 38 had history of some medication intake. Most of the cases due to medicine intake were due to Benzodiazepenes (8 cases) followed by Acetominophen (7), Barbiturates and Anti-histamine (6 each), Aspirin (4), Antidepressant (3), Opiods (2) and Digitalis (2). In the Insecticide group out of 7 subjects, 5 patients were exposed to Organophosphate while 2 patients had ingested Chlorinated Hydrocarbons. Five patients had ingested bathroom cleaners. The mean (std.dev) time interval to reach hospital was 384.25 (894) minutes with median time interval of 60 minutes. Seventy percent of the children reached hospital in less than 180 minutes. Baseline characteristics of patients are shown in table. 1. At the time of presentation most of the children (63%) suffered from dyspnea followed by vomiting in 52% and drowsiness in 51% patients, respectively. Symptomatology of subjects on presentation is shown in table. 2.

**Table 1:** Baseline Characteristics of poisoned patients

Variables	Kerosene oil N = 50 (%)*	Medicine N = 38 (%)*	Insecticide N = 7 (%)*	Bathroom cleaners N = 5 (%)*
**Sex**				
Male	28 (56)	18 (47)	05 (71)	03 (60)
Female	22 (44)	20 (53)	02 (29)	02 (40)
**Living area**				
Urban	40 (80)	35 (92)	05 (71)	05 (100)
Rural	10 (20)	03 (08)	02 (29)	00
**Age (months)**				
≤ 24 months	13 (26)	18 (47)	03 (43)	00
>24 months	37 (74)	20 (53)	04 (57)	05 (100)
Mean ± Std.dev	35 ± 11	32 ± 15	40 ± 18	48 ± 0
				
**Time (mins)**				
≤ 180 mins	40 (80)	25 (66)	05 (71)	00
> 180 mins	10 (20)	13 (34)	02 (29)	05 (100)
Mean ± Std.dev	311 ± 49	205 ± 38	980 ± 64	1560 ± 866
				
**Outcome**				
Discharge	50 (100)	38 (100)	07 (100)	02 (40)
Expired	00	00	00	03 (60)

* All figures have been rounded to the nearest zero; std.dev: standard deviation; mins: minutes.

**Table 2:** Symptomatology observed in subjects on presentation

Variables	Kerosene oil N = 50	Medicine N = 38	Insecticide N = 7	Bathroom Cleaners N = 5
Dyspnea	50	03	05	05
Vomiting	35	05	07	05
Drowsiness	13	30	05	03
Irritability	10	08	00	03
Dilated Pupil	00	00	03	00
Constricted Pupil	00	03	03	03

Of the 50 children in kerosene oil group, 28 were males while 22 were females whereas 18 males and 20 females belonged to medicine group. The mean (std.dev) age in kerosene group was 34.8 (10.4) months where as it was 32.2 (15.4) months in medicine group. Thirty-seven children (74%) were more than 2 years old in kerosene group while 20 children (52.6%) were more than 2 years old in medicine group. Forty out of 50 children (80%) were living in urban area in kerosene group where as 35 out of 38 children (92.1%) were living in urban area in medicine group. The mean (std.dev) time interval to reach hospital in kerosene oil group was 311 (949) minutes and in medicine group was 205 (238) minutes. Forty children (80%) in kerosene group and 25 children (66%) in medicine group reached hospital in less than 3 hours.

All children in kerosene oil, medicine and insecticide groups were discharged satisfactorily, however, 3 children (60%) in bathroom cleaners expired within 48 hours of admission due to respiratory failure.

Storage place of poison was investigated with 76% of parents reporting that it was easily approachable. Education and knowledge of poison in mothers were also evaluated, 73% were illiterate, 19% had high school education whereas only 8% were graduates. Forty-six mothers had sufficient knowledge regarding poison, 33 had none while 21 had some idea about poison and their consequences following ingestion. Majority of the patients (80%) belonged to poor class followed by fair (19%) and only 1 rich in our study. Forty-five patients were having greater than 3 siblings followed by 55 patients who had less than 3 siblings. Data regarding potential risk factors in the household of poisoned children is shown in table. 3.

**Table 3:** Potential risk factors in the household of the subjects

Variable	No. of subjects
**Mothers Education Level**	
Illiterate	73
High School	19
Graduate	08

**Knowledge about Poison**	
Full Awareness	46
Partial Awareness	33
No Awareness	21

**Socioeconomic Status**	
Below Average	80
Average	19
Above Average	01

**No. of Siblings**	
< 3	55
3-5	30
**>**5	15

**Storage Place of Poison**	
Out of approach	24
Easily apprachable	76

## Discussion

Accidental poisoning remains an important health issue in children globally [3] especially in developing countries like Pakistan. This hospital based epidemiological study was carried out in CHK, a tertiary care center catering to both urban and rural areas, where to the best of our knowledge no study of pediatric poisoning has been carried out before. Age, sex ratio, agents responsible and other predisposing risk factors for accidental household poisoning are highlighted in the study.

Male to female ratio in our study was similar to other studies carried all over the world and varies from 1.09-1.8:1 and in our study it was 1.2:1 the same as observed in Saudi study carried out in 1998[7]. In an Indian study the ratio was 1.2:1 due to biological and 1.5:1 due to chemical poisoning[12]. Accidental poisoning occurs mostly in the age group of 0-5 years [12,13] with the peak age between 2-3 years[14-17]. In our study 34% of children affected were under 2 years while 60% were in the range of 2-6 years. Previous studies from Pakistan also show a high prevalence in this age group since children in this age group are more curious leading to injury.

Children belonging to urban areas were more exposed (85%) compared to those in rural areas (15%) this could be due to the advent of careers for mothers due to inflation leading to neglect of child. The agents responsible for childhood poisoning in our study were kerosene oil, medicines, insecticide and bathroom cleaners with kerosene taking first place with 50% cases similar to study carried out in Karachi by Shakya et al, who reported it at 53%[14]. Similar findings have also been observed in other studies carried out previously in developing countries[11,18-20]. Medicine was the second most common agent responsible for poisoning with insecticide and bathroom cleaners following on. The relatively high frequency of these two agents can be attributed to storage of kerosene in coke bottles, a relatively common household practice. Similarly colored medicines like orange and pink attract children[21].

In our study we attempted to find out the quantity of ingested poison by careful history which revealed that 60% of quantity of ingestion was of minor amount and hence of non-toxic nature. In this study 75% of families contacted emergency within 3 hours while 25% contacted after 3 hours due to symptom development this is comparable to study of Saudi Arabia in which 69% sought medical assistance within 2 hours while 31% waited for longer than 4 hours[7]. The common symptomatology was also noted on presentation, In kerosene group dyspnea and vomiting were most common while in medicine group symptoms were different potentially due to the side effects of different medicines intake, similar symptoms have also been observed in accidental poisoning studies carried out in India andTurkey[22,23]. In bathroom cleaner group dyspnea was most common symptom observed. All three fatalities of our study were from the bathroom cleaner group, the subjects had ingested strong acids. The ingestion of strong corrosives resulted in throat burns, edematous vocal cords and respiratory depression. Although ventilation was provided with other life saving measures but despite vigorous efforts the children expired. All patients in bathroom cleaner group presented after 3 hours and it was in the same group that the quantity of poison ingested could not be ascertained.

Poisoning generally is associated with more morbidity than mortality. In studies carried out in India mortality ranged from nil to 11.6% whereas in Pakistan studies it was found to be ranging between 2.5% to13.6%[10,24]. The highest mortality was reported from Karachi in 1982 of 13.6%. Apart from these factors education of mother, their knowledge about poison, storage place of poison along with no. of siblings and no. of family members also contribute to accidental poisoning events. In a multi variant study it was demonstrated that children of young mothers [25,26] and who had only high school education were prone to be have children involved in household poisoning. Similar findings have also been noted in our study. In the same Swedish study, Hjern et al noted that children with more than 2 siblings had a greater chance of all injuries as they got neglected, similar findings were also noted in our study with 45% of the patients having greater than 3 siblings. It was also seen that in 76% of cases poisons were either easily approachable or didn't have proper storage place with medicine kept openly on side tables unlocked and kerosene being kept in kitchen in edible containers[21].

Finally this study fulfills the objective set by the study protocol for this project of assessing the various agents of poisoning and demographic distribution of children brought to CHK with a known history of accidental poison intake and to examine the factors associated with it and advocates the use of mass media campaigns and awareness programs for possible early prevention and management of accidental poisoning cases. This study holds important implications for public health and highlights the high prevalence of accidental household poisoning in the Pakistani population of Asian origin. However there remain certain limitations due to the retrospective nature of the study, moreover due to the small sample size and since CHK receives patients from other cities and rural areas, mainly of low income group this may not represent the true statistics of the area of our study as well as cannot be generalized for the whole population. A large scale prospective multi-center study with appropriate power is recommended for further evaluating the ethnicity, geographic differences and other risk factors for accidental household poisoning in Pakistan.

## Conclusion

On the basis of our study we can conclude that kerosene oil poisoning was the most common with the age group between 2-6 years most commonly involved. The male to female ratio was 1.2:1. Accidental poisoning was the most common in young mothers and in those households having greater than 3 siblings. Literacy rate of mothers correlated significantly with poisoning cases with the highest 73% poisoning cases in children with illiterate mothers. In most of the cases 76% poison was within easy reach. This study highlights the fact that ignorance, neglect and carelessness on part of the parents lead to cases of accidental poisoning.

## Recommendation

On the basis of the study we recommend that:

1) The general warning that 'keep all medicines out of reach of children' should be modified to 'keep all medicines out of reach and sight of children' to suppress their curiosity.

2) Practice of using drink bottles and edible containers for kerosene oil storage should be discouraged.

3) Fully functional poison control centers are the need of the time.

4) Parent counseling and awareness campaigns should be initiated to make parents aware of the hazards of accidental poisoning.

5) Enactment and enforcement of laws for proper packaging of drugs along with the introduction of child resistant packaging and making consumer safety laws more strict.

## Competing interests

The authors declare that they have no competing interests.

## Authors' contributions

NM and SMAS conceived the study, participated in its design and coordination. SMAS and SSF performed the data collection and statistical analysis. NM, BM and SSF drafted the manuscript. BM participated in the design of the study. All the authors read and approved the final manuscript.

## Pre-publication history

The pre-publication history for this paper can be accessed here:

http://www.biomedcentral.com/1471-2431/10/28/prepub
